# Network Pharmacology Exploration Reveals Gut Microbiota Modulation as a Common Therapeutic Mechanism for Anti-Fatigue Effect Treated with Maca Compounds Prescription

**DOI:** 10.3390/nu14081533

**Published:** 2022-04-07

**Authors:** Hongkang Zhu, Ruoyong Wang, Hanyi Hua, Yuliang Cheng, Yahui Guo, He Qian, Peng Du

**Affiliations:** 1 School of Food Science and Technology, Jiangnan University, Wuxi 214122, China; 7210112118@stu.jiangnan.edu.cn (H.Z.); hanyihua@jiangnan.edu.cn (H.H.); ylcheng@jiangnan.edu.cn (Y.C.); guoyahui@jiangnan.edu.cn (Y.G.); 2 Air Force Medical Center, PLA, Beijing 100142, China; 13681118119@163.com

**Keywords:** maca, fatigue, network pharmacology, gut microbiota

## Abstract

Maca compounds prescription (MCP) is a common botanical used in dietary supplements, primarily to treat exercise-induced fatigue. The aim of this study is to elucidate the multi-target mechanism of MCP on fatigue management via network pharmacology and gut microbiota analysis. Databases and literature were used to screen the chemical compounds and targets of MCP. Subsequently, 120 active ingredients and 116 fatigue-related targets played a cooperative role in managing fatigue, where several intestine-specific targets indicated the anti-fatigue mechanism of MCP might be closely related to its prebiotics of intestinal bacteria. Thus, forced swimming tests (FSTs) were carried and mice fecal samples were collected and analyzed by 16S rRNA sequencing. Gut microbiota were beneficially regulated in the MCP-treated group in phylum, genus and OTU levels, respectively, and that with a critical correlation included Lactobacillus and *Candidatus Planktophila*. The results systematically reveal that MCP acts against fatigue on multi-targets with different ingredients and reshapes the gut microbial ecosystem.

## 1. Introduction

Fatigue, best described as an overwhelming feeling of tiredness and exhaustion, affects the quality of life and work efficiency. It is a common and inevitable physiological phenomenon in clinical practice, recently, becoming one of the major threats to human health. Prolonged and recurrent fatigue may lead to aging, palpitation, hypertension, multiple sclerosis, chronic fatigue syndrome (CFS) or even karoshi. Four types of fatigue are generally recognized, namely normal fatigue, episodic fatigue, muscular strain and multiple sclerosis fatigue [1]. Several medicines, such as central nervous system stimulants (which include amphetamine and methylphenidate), can promote wakefulness and alertness, but the side effects of addiction, or even psychotic symptoms cannot be ignored [2]; in particular, the long-term physical health consequences of drug intake could be more serious. Many herbal decoctions have claimed to possess anti-fatigue activity in the Chinese literature, withstanding clinical practice for thousands of years [3]. Herbal medicine exerts its action mainly through regulating the central nervous system, supplementing vital energy and boosting immunity, which could provide additional natural compounds for the management of fatigue [4]. As a famous prescription, Maca compounds prescription (MCP) has been widely used in China, and a diversified chemical composition in MCP is the material basis for its characteristic of acting on multiple targets. Nature medicines always treat fatigue with multiple pharmacological effects by acting as neuroprotective agents, energy supplements, metabolism regulators, antioxidant and inflammatory response inhibitors [4].

Network medicine, as a multidisciplinary science, is a network-based approach to complex diseases with drug targets and biomarkers [5]. According to this, plant-based nutritional and pharmacological treatment modalities can have multi-component, multi-target and multi-pathway effects on anti-fatigue activity [6]. In addition, recent studies reported the vital roles of the gut microbiota in the process of fatigue. Strong evidence shows that bacterial diversity is decreased under a fatigued state [7], whereas targeted supplementation is beneficial to improve mice exercising capacity and intestinal injury via regulating the gut microbiota [8,9]. Recent studies revealed several TCM decoctions could ameliorate fatigue via the gut microbiota, participating in the improvement of the structure of intestinal villi [10], modifying the central nervous system via the gut–brain axis [11]. These findings reveal new strategies of TCM formulations to manage fatigue by targeting the intestinal flora and reshaping the gut microbial ecosystem.

Our previous studies have proved the ameliorative influences of Maca treatment against exercise-induced fatigue via regulating mitochondrial respiration and oxidative stress [12,13]. Meanwhile, MCP has been demonstrated to function in alleviating fatigue, which encouraged our interest in investigating the influence of MCP on the intestinal microbiota, subject to exhaustion induced by acute exercise in the current study. However, limited studies have been found to explain the potential mechanism effect of TCM on the intestinal microbiota, subject to fatigue in mouse models. Hopefully, various multiple activity components and anti-fatigue targets might contribute to alleviating fatigue by irritating the abundance and activity of particular gut bacteria. In this work, based on the bioinformatics methods, we pioneeringly integrate a network pharmacology research strategy, tissue-specific expression analysis and 16S rDNA sequencing, to systematically screen and speculate how TCM formulations regulate exercise-induced fatigue via the targeted gut microbiota in forced swimming mice.

## 2. Materials and Methods

### 2.1. Materials

#### 2.1.1. The Preparation of MCP

The main medicinal parts of plants in MCP were cut into thin sections (3–5 mm). After soaking for 30 min, the medicinal materials were extracted in boiling water (*w*/*v*, 1:8) twice for 1 h. Afterwards, the MCP extract was lyophilized by using a freeze dryer. The lyophilized extract powder was stored at −80 °C for experiments.

#### 2.1.2. The Measurement of the Main Components in MCP

The total polysaccharides of MCP were measured by the phenol-sulfuric acid method, and reducing sugars were assayed with 3,5-dinitrosalicylic acid (DNS). The protein was measured by an automatic nitrogen analyzer (BUCHI), and the total flavonoids in MCP were determined by the aluminum chloride colorimetric method. Amino acid and fatty acid were analyzed by an automatic amino acid analyzer (Agilent 1100, Agilent Technologies Co., Ltd., Santa Clara, CA, USA) and gas chromatography-mass spectrometer (GV2030AF, Shimadzu Co., Ltd., Kyoto, Japan), respectively, as previously described [12].

### 2.2. The Prediction of MCP Targets in Fatigue Using Network Pharmacology

#### 2.2.1. The Screening of Active Ingredients and Potential Targets

We searched the Shanghai Institute of Organic Chemistry of CAS Chemistry Database (http://www.organchem.csdb.cn, 1 January 2022) and literature for all ingredients in Maca, and 26 kinds of macamides were considered as representative constituents of Maca (Table S1) [14]. Swiss Target Prediction (http://www.swisstarget-prediction.ch/, 1 January 2022) was used to predict the targets of macamides. The potential targets of the ingredients can be calculated by entering their SMILES. All the ingredient and target information of the other plants in the MCP were obtained from Traditional Chinese Medicine Systems Pharmacology (TCMSP, http://lsp.nwu.edu.cn/tcmsp.php, 1 January 2022) and Traditional Chinese Medicine Integrated Database (TCMID, http://119.3.41.228:8000/tcmid/, 1 January 2022). The active ingredients of the MCP were screened based on oral bioavailability (OB) ≥ 30% and drug-likeness (DL) ≥ 0.18. Additionally, chemical constituents with anti-fatigue-related pharmacological activity were also selected as potential ingredients according to previous literature investigations. The Universal Protein (UniProt, http://www.UniProt.org, 1 January 2022) database was used to standardize all the targets [15].

#### 2.2.2. Prediction of Anti-Fatigue Targets of the MCP

Online Mendelian Inheritance In Man (OMIM, http://www.ncbi.nlm.nih.gov/omim, 1 January 2022), Therapeutic Target Database (TTD, http://db.idrblab.net/ttd/, 1 January 2022), Drugbank (https://go.drugbank.com/, 1 January 2022) and Gene Cards (http://www.genecards.org/, Relevance score ≥ 10, 1 January 2022) databases were used to search and screen the known fatigue targets for the subsequent study. Target information from the four databases were combined and duplicates were removed. The intersection of the fatigue targets and ingredient targets is considered as a potential target of the MCP in treating fatigue.

#### 2.2.3. The Construction of Active Ingredients: Target Network

The active ingredients in the MCP and their corresponding targets were imported into Cytoscape 3.8.0 to construct an active ingredient-target network.

#### 2.2.4. The Construction of the Protein Interaction Network

The obtained potential targets were entered into the STRING (https://string-db.org, 1 January 2022) database, and “Homo sapiens (Human), 9606” proteins with the minimum required interaction score higher than 0.900 were picked out. The targets and their combined scores were imported into Cytoscape 3.8.0 to construct a protein interaction network. The node size reflects the node degree, where the higher the degree value, the larger the node size. Meanwhile, the node color is proportional to the degree interactions (red indicates up-regulation, and blue indicates down-regulation).

#### 2.2.5. Tissue-Specific Gene Expression Analysis

To screen out tissue-specific targets in the intestine (small intestine and colon), the human protein atlas (https://www.proteinatlas.org, 1 January 2022) was used to download 887 elevated genes, which show some level of elevated expression in the intestine compared to other tissues. The intersection of those two gene sets yielded the intestine-specific anti-fatigue targets of the MCP. The protein expression data from the intestine are derived from antibody-based profiling using immunohistochemistry.

#### 2.2.6. Functional Enrichment Analysis

The identified targets were transformed to Gene ID by using DAVID Bioinformatics Resources 6.8 (https://david.ncifcrf.gov/home.jsp, 1 January 2022). Then, the functional enrichment analysis was analyzed the Gene Ontology (GO) and Kyoto Encyclopedia of Genes and Genomes (KEGG) enrichment, and the bar and plot graphs were created for the visualization of these enrichment results.

### 2.3. Gut Microbiota Composition in Swimming Mice

#### 2.3.1. Experimental Animals

All experimental animal procedures were approved by the Ethics Committee of Experimental Animal Center of Jiangnan University (JN.No20200710i0600925). The experiments were performed when ICR mice (18–22 g) had adapted to the experimental environment for a week. Animals were randomly divided to 2 different groups: orally administrated with saline (Control, Con, *n* = 10), or the MCP (2.0 g/kg.bw., *n* = 10) for 30 days.

#### 2.3.2. Weight-Loaded Forced Swimming Test (WFST)

Half of the mice in the Con and MCP groups (*n* = 5) were selected at random and were exposed to an exhaustive swimming test, which was carried out 1 h after the oral administration of saline or the MCP on the last day. The mice were placed in a pool with room temperature water (25 ± 2 °C). The mice were loaded with a lead block (5% of bw.) attached to their tails. The mice were judged as exhausted when they sunk into the water for 7 s and failed to return to the surface of water. The exhaustion time was recorded to evaluate the mice swimming capacity.

#### 2.3.3. 16S rRNA Gene and Bioinformatics Analysis

The other 5 mice fecal samples were collected and DNA from the mice feces was extracted by using a Genomic DNA Kit (Omega Bio-tek, Inc., Norcross, GA, USA), as previously stated [16,17]. The 16S rRNA genes (V3-V4 regions) were amplified from the whole genome via the primer pair (341 F, 5′- CCTAYGGGRBGCASCAG-3′; 806R, 5′-GGACTACHVGGGTWTCTAAT-3′) with the barcode. All the amplicons were purified, quantified, and then sequenced on an Illumina novaseq platform and 250 bp paired-end reads were generated. The barcode and connector sequence were cleared away, and the double-ended sequences were stitched by using FLASH (v1.2.8) and the unqualified sequences were filtered out. Finally, the sequence with 97% similarity was classified as an OTU, and the systemic affinities of the ITS2 gene sequence of RDP and Unite database were used for distributing the 16S rRNA genes into distinct taxonomic categories.

### 2.4. Data Analysis

GraphPad Prism 7 was used for analysis. The results were expressed as the mean ± standard deviation. One-way ANOVA with Dennett’s multiple comparisons test was used for the comparison between the three groups; * *p* < 0.05, ** *p* < 0.01 were statistically significant among the groups. Microbiological analyses and structural equation modeling analyses were conducted by using R software version 4.1.0. Principal component analysis was conducted by using the ggpubr package, hierarchical clustering was performed by using Cluster, and the presented heatmap plot was generated using the R heatmap package.

## 3. Results and Discussion

### 3.1. Measurement of the MCP Components

**Figure 1:**
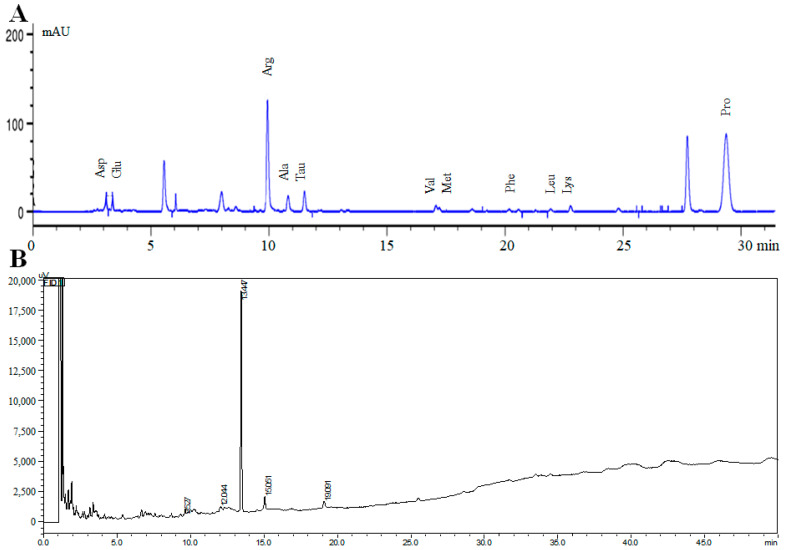
Liquid chromatogram of the free amino acids (**A**) and gas chromatogram analysis of free fatty acid composition (**B**) in the MCP.

For general nutrition, the MCP contains 34.78 mg/mL of total polysaccharides, 8.64 mg/mL of reducing sugar, 0.812 mg/mL of total proteins, 0.157 mg/mL of flavonoids, 18 types of amino acids and 9 types of fatty acids, as shown in Table 1. The MCP consists of rich amino acids, including fatigue-related amino acid—taurine (Tau, 98.615 μg/mL). Among them, 767.99 μg/mL of arginine (Arg) and 548.88 μg/mL of proline (Pro) were at higher levels than other amino acids (Table 2). For fatty acids, the MCP mainly contains pentadecanoic acid (C15:0, 95.20 μg/mL), which played a vital role in energy metabolism and antioxidant defenses in the muscles [17]. The chromatographic fingerprints are shown in Figure 1.

### 3.2. Network Pharmacology Analysis of the Main Components of the MCP

**Figure 2:**
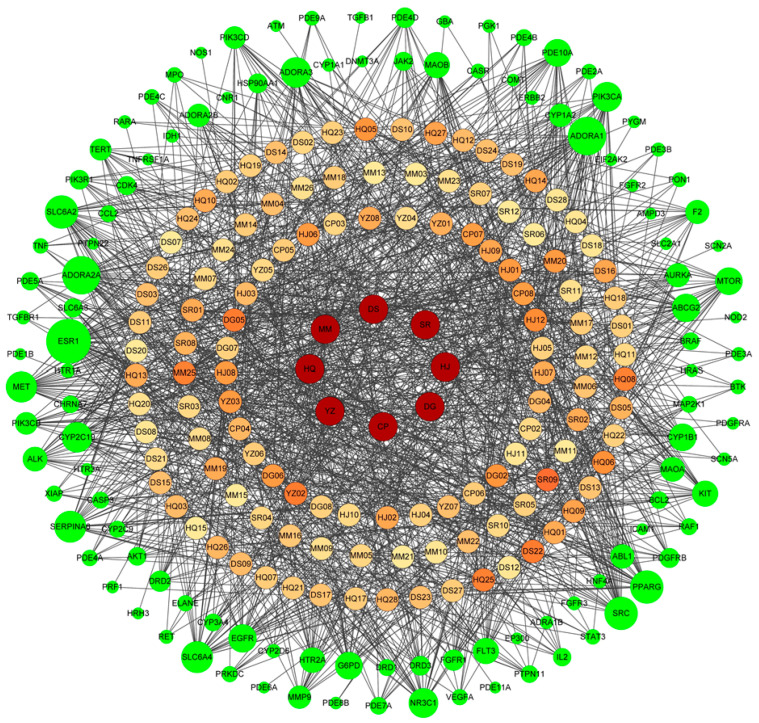
The single herb-active ingredients–target network. The center blue circle represents 8 single herbs, the orange circle represents the active ingredients in the MCP, and the green circle represents the targets. The shades of color and the size of the nodes represent the degrees of active ingredients and targets, respectively.

The exhaustion swimming time of mice treated with the MCP showed significant increases compared to the Con group (25.1 min vs. 10.5 min, *p* < 0.01). Using the established filter conditions OB ≥ 30% and DL ≥ 0.18, 26 macamides (Table S1) and 120 active ingredients were identified in the MCP (Table S2). All the fatigue-related targets were combined and 438 targets were obtained as a merged set. After matching the MCP active ingredient targets with the fatigue-related targets, a total of 116 potential anti-fatigue targets for the MCP were obtained (Table S3). The MCP active ingredients–target network diagram was constructed based on the interactions among the 8 herbs, 120 active ingredients and 116 fatigue-related targets (Figure 2). Of the 8 herbs, Huangqi has 27 active ingredients with 327 targets, and Dangshen has 25 active ingredients with 216 targets, and 26 macamides in Maca have 166 targets. Of the 120 active ingredients, 10 of them have more than 20 targets, for example, vitamin E (SR06, degree = 28), phyllanthin (DG05, degree = 26), 7-Methoxy-2-methyl isoflavone (DS22, degree = 26), 4’,5,7-trihydroxy-6-methyl-8-methoxy-homoisoflavanone (YZ02, degree = 26), 3,9-di-O-methylnissolin (HQ08, degree = 25), medicarpin (HQ25, degree = 25), and N-benzyl-9-oxo-10E,12Z-octadecadienamide (MM25, degree = 25) had a high degree value. From the perspective of the targets, 23 targets worked with more than 20 ingredients (Table S3). The top 5 targets were estrogen receptor 1 (ESR1), adenosine A1 receptor (ADORA1), adenosine A2a receptor (by homology) (ADORA2A), tyrosine protein kinase (SRC), and norepinephrine transporter (SLC6A2), which interacted with 47, 38, 38, 31 and 31 ingredients, respectively. As shown in Figure 2, we found that some ingredients in the MCP could act on multiple targets, while various ingredients could work together on the same target, reflecting the mechanism of interaction between “multiple ingredients” and “multiple targets” based on the TCM theory.

### 3.3. PPI Core Network Analysis and Specificity of Targets in Tissue Expression

**Figure 3:**
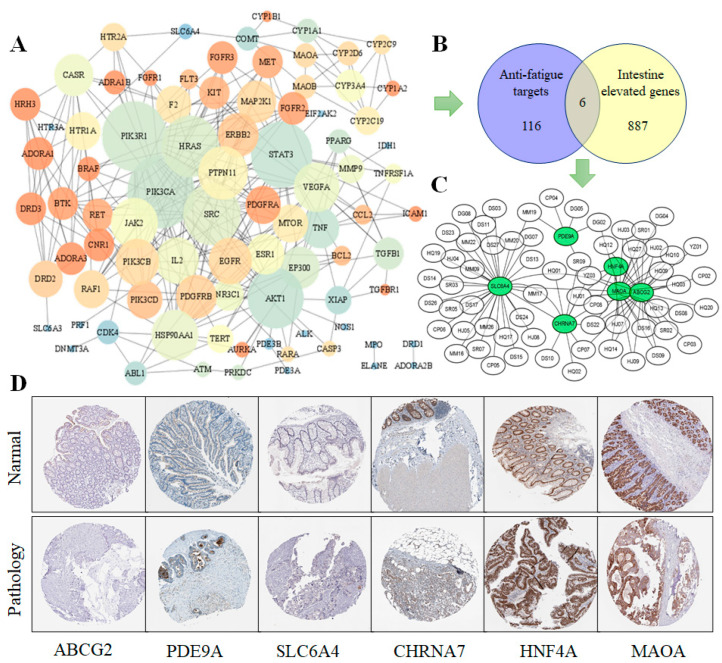
Action view of the interaction network obtained from STRING. (**A**) PPI network of 86 targets and 340 edges. (**B**) Venn diagram of anti-fatigue targets and intestine elevated genes. (**C**) The subnetworks of six targets: ABCG2, PDE9A, SLC6A4, CHRNA7, HNF4A and MAOA. (**D**) Immunohistochemistry staining of the 6 intestine-specific expression targets in normal and pathology tissues.

Using 116 anti-fatigue targets of the MCP entered into the String database to obtain the protein interaction data, the Cytoscape 3.8.0 software was used to map these targets to the human protein–protein interaction network; a total of 86 targets and 340 edges (combined score ≥ 0.9) were obtained (Figure 3A). Then, the specificity of the targets in intestine expression were obtained from The Human Protein Atlas. The 116 anti-fatigue targets of the MCP and the 887 intestine elevated genes were used to draw a Venn diagram; 6 targets were obtained for both (Figure 3B), which were preferentially expressed in the intestine. The results show that the MCP plays a cooperative role in managing fatigue through multiple potential targets in the intestine. The intestine-specific expression of the markers of the anti-fatigue targets are ABCG2 (small intestine), PDE9A (colon), SLC6A4 (colon), CHRNA7 (colon), HNF4A (colon) and MAOA (small intestine and colon). The subnetworks of the six intestine-specific targets were extracted (Figure 3C). Most of the ingredients could work together on the target of SLC6A4, while ABCG2, HNF4A and MAOA shared several same ingredients, indicating similar biological functions. For further research, immunohistochemistry staining of the 6 intestine-specific expression targets (Figure 3D) was obtained from the Human Protein Atlas database, showing that these proteins were differentially expressed between pathology tissues (colorectal cancer) and normal colon samples (Figure 3D). Meanwhile, the difference of intestinal morphology indicates its potential role in gut functions, such as the intestinal barrier, gut-metabolism, or permeability. In summary, it is very likely that the anti-fatigue mechanism of the MCP is closely related to its vital roles in intestinal bacteria and the overall health of the host.

### 3.4. Improvement on the Bacterial Diversity and Richness of Mice Gut Microbiota

**Figure 4:**
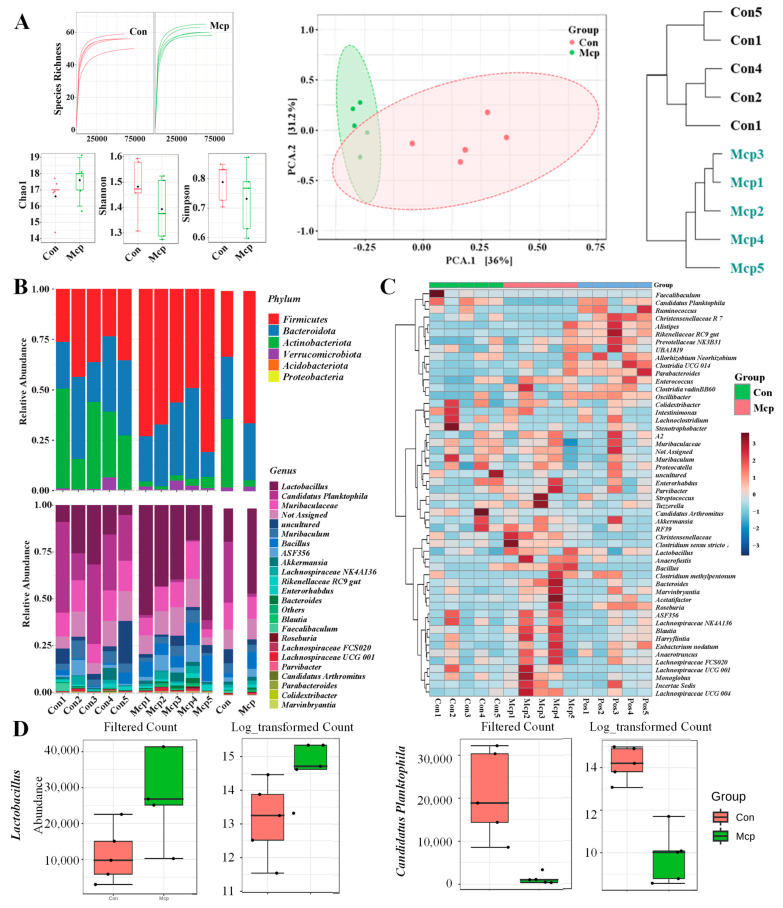
Changes and differences in the gut microbiota. (**A**) Effect of the MCP on species richness and the abundance of gut microbiota; (**B**) phylum and genus level; (**C**) OTU level; and (**D**) the relative abundance of *Lactobacillus* and *Candidatus Planktophila* on the genus levels (red boxplots represent the Con group and the green represent the MCP group).

In order to illustrate the abundance of species in gut microbiota, a curve was constructed between the number of sequences obtained by random sampling and the number of corresponding OTUs. It can be seen from Figure 3A that the MCP group had a higher index among all the tested samples, indicating higher richness of bacterial communities. Additionally, to reveal whether the MCP affected diversity, we further analyzed the alpha diversity of all the samples. The total number of species can be represented by Chao1, and Shannon and Simpson indexes representing the gut microbial diversity confirmed the microbial diversity [18]. The results show that the MCP can change the gut microbiome in mice. The results of the beta diversity, including principal component analysis (PCA) and cluster tree, found that the MCP group was distinguished from the Con group, further suggesting the effects of the MCP on the composition of the microbiota community. Microbiota in the gut includes predominant phylum, *Bacteroides*, *Firmicutes*, and *Verrucomicrobia*. As shown in Figure 4B, there are notable changes in the relative abundance of phylum: *Firmicutes* and *Actinobacteriota*. Compared to the Con group, the relative abundance of *Actinobacteriota* in the MCP groups decreased, while that of *Firmicutes* increased, demonstrating that the MCP markedly boosts *Firmicutes*, and inhibits *Actinobacteriota*. To determine the changes of microbial community at the OTU level after the administration of the MCP, a correlation between the key OTUs was exhibited by a heat map (Figure 4C). To further reveal the specific genus in metabolizing the MCP, the genus in the samples were analyzed. As a result, the relative abundance of *Lactobacillus* was significantly increased (*p* = 0.017) with the intervention of the MCP, while that of *Candidatus Planktophila* significantly decreased (*p* = 4.94 × 10^−5^) (Figure 4D). Several studies found *Lactobacillus*, as ingested microorganism probiotics, can significantly relieve fatigue, which may increase muscle mass, energy harvesting, exercise performance and have health-promotion effects [19,20,21]. *Candidatus Planktophila* is an actinobacterium representing one of the most numerically important taxa in freshwater bacterioplankton, which may be pathogens from the environment [22]. Given that gut microbiota is significantly associated with energy expenditure and host metabolism, these findings suggest a potential central role of the gut microbiota in mediating the anti-fatigue effect of the MCP. It indicated that the MCP could probably serve as a prebiotic and beneficially regulate the gut microbiota and the reshaped gut microbial ecosystem.

### 3.5. GO and KEGG Enrichment Analysis

**Figure 5:**
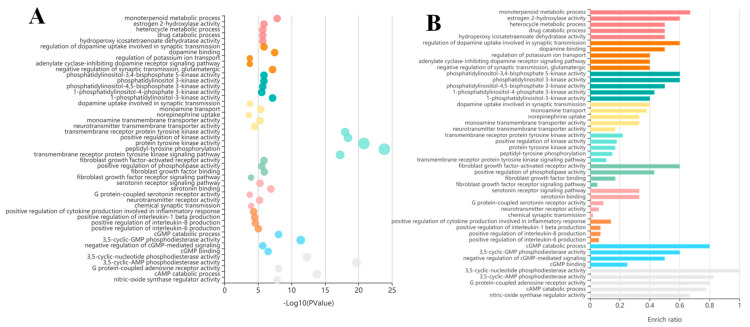
Gene Ontology (GO) and Kyoto Encyclopedia of Genes and Genomes (KEGG) enrichment. (**A**) Biological process enrichment analysis for the effect of the MCP on fatigue. (**B**) Signal pathway enrichment analysis for the effect of the MCP on fatigue.

To further elucidate the possible anti-fatigue effects of the MCP, the biological processes and signaling pathways of 116 key targets were carried out through the gene enrichment analysis. The results show that the biological processes (*p* < 0.05) are largely related to the monoterpenoid metabolic process, the regulation of dopamine uptake involved in synaptic transmission and phosphatidylinositol-3,4-bisphosphate 5-kinase activity (Figure 5A), and the signaling pathways (*p* < 0.05) were mainly involved in the central carbon metabolism in cancer, type II diabetes mellitus and the TNF signaling pathway (Figure 5B).

## 4. Conclusions

Chinese medicine has a long history, focusing on the overall improvement of the entire body and resistance to endogenous and exogenous damage. Network pharmacology is considered to be a comprehensive approach to discovering the bioactive ingredients in and action mechanisms of the TCM [23]. In this study, network pharmacology analysis predicted the anti-fatigue effect of the MCP through multiple potential targets involving 120 active ingredients and 116 fatigue-related targets. Previous studies showed that vitamin E was widely recognized among athletes as a possible method for enhancing athletic performance [24]. As an antioxidant, vitamin E blunts the mRNA expression of ROS-related, inflammatory, and mitochondria proteins in cellular signaling in skeletal muscle [25]. According to the literature reports, the other key compounds also have plentiful biological activities. Phyllanthin [26], 7-Methoxy-2-methyl isoflavone [27], medicarpin [28], and N-benzyl-9-oxo-10E,12Z-octadecadienamide [29] displayed anti-inflammatory, anti-oxidant, neuroprotective, and anti-cancer effects. These studies suggest that the MCP has strong support in fatigue management via network pharmacology.

Recently, an exploratory study indicated that gut microbiota and diet might be related to mental and physical energy and fatigue traits [30]. Meanwhile, dietary supplements were reported to lead to an improvement in exercise performance and a resistance of physical fatigue via the alteration of the gut microbiota composition [31,32]. Thus, dietary intervention therapeutically targeting the microbiota may need to be incorporated in diets of motor populations to combat exercise-induced fatigue. Several intestine-specific expressed targets are essential to adjust intestinal health, such as ABCG2, which modulates the absorption of drugs in the intestine via affecting their bioavailability [33]. Our results verify that a one-month administration of the MCP enhances mice exercise capacity and relieves fatigue via enhancing the abundance and activity of particular gut bacteria, such as *Firmicutes* and *Actinobacteriota*. Furthermore, the relative abundance of Lactobacillus increases with the intervention of the MCP, while that of *Candidatus Planktophila* has a significant decrease. The 16S rRNA gene and bioinformatics analysis highlighted that the MCP was beneficial to the gut micro-ecological balance in the exercise mice, indicating that the MCP may be used as a prebiotic to prevent exercise-induced fatigue.

Therefore, fatigue may be a consequence of gastrointestinal imbalance, and targeting on specific intestinal bacteria and gut microbial ecosystem modulation may be a worthwhile therapeutic strategy in fatigue management [9,34]. Although such associations between gut microbiota and the MCP are established, the underlying mechanisms by which the microbiota and the host interact remain unknown. Therefore, further explorations of the host-microbe-drug-nutrient is required to address the fundamental question of how gut microbes and nutrition, key regulators of host physiology, affect the effects of the MCP [35].

In conclusion, the MCP has a potential therapeutic effect on the management of fatigue according to the network pharmacology and gut microbiota analysis.

## Figures and Tables

**Table 1:** Major bioactive components in the MCP.

Major Bioactive Components	Contents
Total polysaccharides (mg/mL)	34.78
Reducing sugar (mg/mL)	8.64
Total proteins (mg/mL)	0.812
Total amino acids (mg/mL)	1845.27
Total fatty acids (μg/mL)	110.59
Total flavonoids (mg/mL)	0.157

**Table 2:** The composition of amino acids and fatty acids in the MCP.

	Amino Acids	Ret. Time (min)	Peak Area (mAU`S)	Contents (μg/mL)
**Amino Acids**	Asp	3.123	267.597	3.486
	Glu	3.377	133.185	4.081
	Ser	6.403	3.658	2.706
	His	7.342	82.776	5.315
	Gly	8.303	51.938	1.955
	Thr	8.614	267.514	3.230
	Arg	9.943	3420.104	4.611
	Ala	10.820	144.076	2.256
	Tau	11.503	466.864	3.310
	Tyr	13.076	26.769	5.064
	Cys	16.360	6.677	4.948
	Val	17.056	34.216	3.037
	Met	17.50	11.686	3.760
	Trp	19.216	19.450	6.350
	Phe	20.175	36.052	4.441
	Ile	20.552	46.827	3.363
	Leu	21.916	53.758	3.335
	Lys	22.752	211.235	2.224
	Pro	29.295	8255.328	2.010
**Fatty Acids**	C12:0	9.527	828	0.769
	C14:0	12.044	3292	3.059
	C15:0	13.447	92632	86.08
	C16:0	15.051	6117	5.685
	C18:0	19.091	4742	4.407

## Data Availability

Not applicable.

## Supplementary Materials

The following supporting information can be downloaded at: https://www.mdpi.com/article/10.3390/nu14081533/s1, Table S1: 26 kinds of macamides from Maca, Table S2: Potential active ingredients of Maca compound preparation, Table S3: Target information of potential active ingredients of Maca compound.
