# Supplementary material for: Network Pharmacology Exploration Reveals Gut Microbiota Modulation as a Common Therapeutic Mechanism for Anti-Fatigue Effect Treated with Maca Compounds Prescription
Source: Nutrients. 2022 Apr 7;14(8):1533. doi: 10.3390/nu14081533 (PMC9026883; doi:10.3390/nu14081533)
Supplement: Supplementary file 1 [file nutrients-14-01533-s001.zip › nutrients-1605486-supplementary.pdf]

Table S1 26 kinds of macamides from Maca

No.	Name	Formula	SMILE
MM01	N-benzyl-9,16-dioxo-10E,12E,14E-octadecatrienamide	$C_{25}H_{33}NO_3$	CCC(=O)\C=C\C=C\C=C\C(=O)CCCCCCCC(=O)NCC1=CC=CC=C1
MM02	N-benzyl-16(S)-hydroxy-9-oxo-10E,12E,14E-octadecatrienamide	$C_{25}H_{35}NO_3$	CC[C@H](O)\C=C\C=C\C=C\C(=O)CCCCCCCC(=O)NCC1=CC=CC=C1
MM03	N-benzyl-5-oxo-6E,8E-octadecadienamide	$C_{25}H_{37}NO_2$	CCCCCCCCC\C=C\C=C\C(=O)CCCC(=O)NCC1=CC=CC=C1
MM04	N-benzyl-hexadecanamide	$C_{23}H_{39}NO$	CCCCCCCCCCCCCCCC(=O)NCC1=CC=CC=C1
MM05	N-benzyl-9-oxo-12Z-octadecenamide	$C_{25}H_{39}NO_2$	CCCCC\C=C\CCC(=O)CCCCCCCC(=O)NCC1=CC=CC=C1
MM06	N-benzyl-9-oxo-12Z,15Z-octadecadienamide	$C_{25}H_{37}NO_2$	CC\C=C/C\C=C/CCC(=O)CCCCCCCC(=O)NCC1=CC=CC=C1
MM07	N-benzyl-13-oxo-9E,11E-octadecadienamide	$C_{25}H_{37}NO_2$	CCCCCC(=O)\C=C\C=C\CCCCCCCC(=O)NCC1=CC=CC=C1
MM08	N-benzyl-15Z-tetracosenamide	$C_{31}H_{53}NO$	CCCCCCCC\C=C/CCCCCCCCCCCCCC(=O)NCC1=CC=CC=C1
MM09	N-(m-methoxybenzyl)-hexadecanamide	$C_{24}H_{41}NO_2$	CCCCCCCCCCCCCCCC(=O)NCC1=CC(OC)=CC=C1
MM10	N-benzyl-9Z-octadecenamide	$C_{25}H_{41}NO$	CCCCCCCC\C=C\CCCCCCCC(=O)NCC1=CC=CC=C1
MM11	N-(m-methoxybenzyl)-9Z-octadecenamide	$C_{26}H_{43}NO_2$	CCCCCCCC\C=C\CCCCCCCC(=O)NCC1=CC(OC)=CC=C1
MM12	N-benzyl-9Z,12Z-octadecadienamide	$C_{25}H_{39}NO$	CCCCC\C=C\C\C=C\CCCCCCCC(=O)NCC1=CC=CC=C1
MM13	N-(m-methoxybenzyl)-9Z,12Z-octadecadienamide	$C_{26}H_{41}NO_2$	CCCCC\C=C\C\C=C\CCCCCCCC(=O)NCC1=CC(OC)=CC=C1
MM14	N-benzyl-9Z,12Z,15Z-octadecatrienamide	$C_{25}H_{37}NO$	CC\C=C\C\C=C\C\C=C\CCCCCCCC(=O)NCC1=CC=CC=C1
MM15	N-(m-methoxybenzyl)-9Z,12Z,15Z-octadecatrienamide	$C_{26}H_{39}NO_2$	CC\C=C\C\C=C\C\C=C\CCCCCCCC(=O)NCC1=CC(OC)=CC=C1
MM16	N-benzyl-octadecanamide	$C_{25}H_{43}NO$	CCCCCCCCCCCCCCCCCC(=O)NCC1=CC=CC=C1
MM17	N-benzyl-pentadecanamide	$C_{22}H_{37}NO$	CCCCCCCCCCCCCCC(=O)NCC1=CC=CC=C1
MM18	N-benzyl-heptadecanamide	$C_{24}H_{41}NO$	CCCCCCCCCCCCCCCCC(=O)NCC1=CC=CC=C1
MM19	N-benzyl-octanamide	$C_{15}H_{23}NO$	CCCCCCCC(=O)NCC1=CC=CC=C1
MM20	N-(m-methoxybenzyl)-octanamide	$C_{16}H_{25}NO$	CCCCCCCC(=O)NCC1=CC(OC)=CC=C1
MM21	N-(3,4-dimethoxybenzyl)-hexadecanamide	$C_{25}H_{43}NO_3$	CCCCCCCCCCCCCCCC(=O)NCC1=CC(OC)=C(OC)C=C1

MM22	N-benzyl-tetracosanamide	$C_{31}H_{55}NO$	CCCCCCCCCCCCCCCCCCCCCCC C(=O)NCC1=CC=CC=C1
MM23	N-benzyl-2E-octadecadienamide	$C_{25}H_{41}NO$	CCCCCCCCCCCCCCC\C=C\C(=O)N CC1=CC=CC=C1
MM24	N-benzyl-9-oxo-10E,12E-octadecadienamide	$C_{25}H_{37}NO_2$	CCCCC\C=C\C=C\C(=O)CCCCCCC C(=O)NCC1=CC=CC=C1
MM25	N-benzyl-9-oxo-10E,12Z-octadecadienamide	$C_{25}H_{37}NO_2$	CCCCC\C=C/C=C/C(=O)CCCCCCC C(=O)NCC1=CC=CC=C1
MM26	N-(3,4-dimethoxybenzyl)-9Z-oleamide	$C_{27}H_{45}NO_3$	CCCCCCCC\C=C/CCCCCCCC(=O)N CC1=CC(OC)=C(OC)C=C1

Table S2 Potential active ingredients of Maca compound preparation

	No.	Chemical composition	Molecular	OB%	DL%
Citrus reticulata Blanco (Chenpi)	CP01	carotene	$C_{40}H_{56}$	37.18	0.58
	CP02	citromitin	$C_{21}H_{24}O_8$	86.90	0.51
	CP03	nobiletin	$C_{27}H_{32}O_{14}$	61.67	0.52
	CP04	obacunone	$C_{26}H_{30}O_7$	81.58	0.57
	CP05	β-sitosterol	$C_{29}H_{50}O$	33.94	0.70
	CP06	sitosterol	$C_{29}H_{50}O$	36.91	0.75
	CP07	5,7-dihydroxy-2-(3-hydroxy-4-methoxyphenyl)chroman-4-one	$C_{16}H_{14}O_6$	47.74	0.27
	CP08	naringenin	$C_{15}H_{12}O_5$	59.29	0.21
Angelica sinensis (Danggui)	DG01	chrysanthemaxanthin	$C_{40}H_{56}O_3$	38.72	0.58
	DG02	cnidilin	$C_{17}H_{16}O_5$	32.69	0.28
	DG03	folinicacid	$C_{20}H_{23}N_7O_7$	31.79	0.74
	DG04	isoimperatorin	$C_{16}H_{14}O_4$	45.46	0.23
	DG05	phyllanthin	$C_{24}H_{34}O_6$	33.31	0.42
	DG06	suchilactone	$C_{21}H_{20}O_6$	57.52	0.56
	DG07	beta-sitosterol	$C_{29}H_{50}O$	36.91	0.75
	DG08	Stigmasterol	$C_{29}H_{48}O$	43.83	0.76
Codonopsis pilosula (Dangshen)	DS01	5alpha-stigmastan-3,6-dione	$C_{29}H_{48}O_2$	33.12	0.79
	DS02	5α-stigmasta-7,22-dien-3-one	$C_{29}H_{46}O$	42.98	0.76
	DS03	alpha-spinasterol	$C_{29}H_{48}O$	42.98	0.76
	DS04	chrysanthemaxanthin	$C_{40}H_{56}O_3$	38.72	0.58
	DS05	daturilin	$C_{28}H_{36}O_4$	50.37	0.77
	DS06	diisocapryl phthalate	$C_{24}H_{38}O_4$	43.59	0.39
	DS07	friedelin	$C_{30}H_{50}O$	29.16	0.76
	DS08	frutinone a	$C_{16}H_8O_4$	65.9	0.34
	DS09	glycitein	$C_{16}H_{12}O_5$	50.48	0.24
	DS10	methyl octadecadienoate	$C_{19}H_{34}O_2$	41.93	0.17
	DS11	stigmasta-5	$C_{29}H_{48}O$	36.91	0.75
	DS12	stigmasta-5,22-dien-3-one	$C_{29}H_{46}O$	43.83	0.76
	DS13	taraxerol	$C_{30}H_{50}O$	38.4	0.77

	DS14	α-spinasterol	$C_{29}H_{48}O$	42.98	0.76
	DS15	3-beta-Hydroxymethyllenetanshiquinone	$C_{18}H_{14}O_4$	32.16	0.41
	DS16	luteolin	$C_{15}H_{10}O_6$	36.16	0.25
	DS17	stigmast-7-enol	$C_{29}H_{50}O$	37.42	0.75
	DS18	7-(beta-Xylosyl)cephalomannine_qt	$C_{43}H_{53}O_{13}N$	38.33	0.29
	DS19	methyl icosa-11,14-dienoate	$C_{21}H_{38}O_2$	39.67	0.23
	DS20	Spinoside A	$C_{39}H_{56}O_{12}$	39.97	0.4
	DS21	11-Hydroxyrankinidine	$C_{18}H_{22}O_4N_2$	40	0.66
	DS22	7-Methoxy-2-methyl isoflavone	$C_{17}H_{14}O_3$	42.56	0.2
	DS23	poriferasta-7,22E-dien-3beta-ol	$C_{29}H_{48}O$	42.98	0.76
	DS24	Spinasterol	$C_{29}H_{48}O$	42.98	0.76
	DS25	Diop	$C_{24}H_{38}O_4$	43.59	0.39
	DS26	ZINC03978781	$C_{29}H_{48}O$	43.83	0.76
	DS27	Stigmasterol	$C_{29}H_{48}O$	43.83	0.76
	DS28	(8S,9S,10R,13R,14S,17R)-17-[(E,2R,5S)-5-ethyl-6-methylhept-3-en-2-yl]-10,13-dimethyl-1,2,4,7,8,9,11,12,14,15,16,17-dodecahydrocyclopenta[a]phenanthren-3-one	$C_{29}H_{46}O$	45.4	0.76
Polygonatum sibiricum Red. (Huangjing)	HJ01	DFV	$C_{15}H_{12}O_4$	32.76	0.18
	HJ02	baicalein	$C_{15}H_{10}O_5$	33.52	0.21
	HJ03	3'-Methoxydaidzein	$C_{16}H_{12}O_5$	48.57	0.24
	HJ04	beta-sitosterol	$C_{29}H_{50}O$	36.91	0.75
	HJ05	sitosterol	$C_{29}H_{50}O$	36.91	0.75
	HJ06	methylprotodioscin_qt	$C_{28}H_{46}O_3$	35.12	0.86
	HJ07	(2R)-7-hydroxy-2-(4-hydroxyphenyl)chroman-4-one	$C_{15}H_{12}O_4$	71.12	0.18
	HJ08	diosgenin	$C_{27}H_{42}O_3$	80.88	0.81
	HJ09	4',5-Dihydroxyflavone	$C_{15}H_{10}O_4$	48.55	0.19
	HJ10	sibiricoside A_qt	$C_{27}H_{44}O_4$	35.26	0.86
	HJ11	Syringaresinol-O-beta-D-glucoside	$C_{28}H_{36}O_{13}$	43.35	0.77
	HJ12	zhonghualiaoine 1	$C_{29}H_{48}O_3$	34.72	0.78
Astragalus membranaceus (Huangqi)	HQ01	isoflavanone	$C_{16}H_{14}O_6$	109.99	0.3
	HQ02	7-O-methylisomucronulatol	$C_{18}H_{20}O_5$	74.69	0.3
	HQ03	formononetin	$C_{16}H_{12}O_4$	69.67	0.21
	HQ04	FA	$C_{19}H_{19}N_7O_6$	68.96	0.71
	HQ05	(3R)-3-(2-hydroxy-3,4-dimethoxyphenyl)chroman-7-ol	$C_{17}H_{18}O_5$	67.67	0.26
	HQ06	(6aR,11aR)-9,10-dimethoxy-6a,11a-dihydro-6H-benzofurano[3,2-c]chromen-3-ol	$C_{17}H_{16}O_5$	64.26	0.42
	HQ07	Mairin	$C_{30}H_{48}O_3$	55.38	0.78
	HQ08	3,9-di-O-methylnissolin	$C_{18}H_{18}O_5$	53.74	0.48
	HQ09	Jaranol	$C_{17}H_{14}O_6$	50.83	0.29
	HQ10	isorhamnetin	$C_{16}H_{12}O_7$	49.6	0.31
	HQ11	isomucronulatol-7,2'-di-O-glucosiole	$C_{29}H_{38}O_{15}$	49.28	0.62

	HQ12	Calycosin	$C_{16}H_{12}O_5$	47.75	0.24
	HQ13	quercetin	$C_{15}H_{10}O_7$	46.43	0.28
	HQ14	kaempferol	$C_{15}H_{10}O_6$	41.88	0.24
	HQ15	5'-hydroxyiso-muronulatol-2',5'-di-O-glucoside	$C_{29}H_{38}O_{16}$	41.72	0.69
	HQ16	1,7-Dihydroxy-3,9-dimethoxy pterocarpene	$C_{17}H_{14}O_6$	39.05	0.48
	HQ17	hederagenin	$C_{29}H_{50}O$	36.91	0.75
	HQ18	9,10-dimethoxypterocarpan-3-O-β-D-glucoside	$C_{23}H_{28}O_{10}$	36.74	0.92
	HQ19	(3S,8S,9S,10R,13R,14S,17R)-10,13-dimethyl-17-[(2R,5S)-5-propan-2-yloctan-2-yl]-2,3,4,7,8,9,11,12,14,15,16,17-dodecahydro-1H-cyclopenta[a]phenanthren-3-ol	$C_{30}H_{52}O$	36.23	0.78
	HQ20	Bifendate	$C_{20}H_{18}O_{10}$	31.1	0.67
	HQ21	astragalosideI_qt	$C_{39}H_{62}O_{11}$	12.34	0.2
	HQ22	astragalosideII_qt	$C_{37}H_{60}O_{10}$	11.55	0.25
	HQ23	astragalosideIV_qt	$C_{35}H_{58}O_9$	7.07	0.32
	HQ24	astragalosideIII_qt	$C_{35}H_{58}O_9$	5.35	0.32
	HQ25	medicarpin	$C_{16}H_{14}O_4$	49.22	0.34
	HQ26	chrysanthemaxanthin	$C_{40}H_{56}O_3$	38.72	0.58
	HQ27	kumatakenin	$C_{17}H_{14}O_6$	50.83	0.29
	HQ28	β-sitosterol	$C_{27}H_{30}O_{12}$	33.94	0.7
	SR01	meletin	$C_{15}H_{10}O_7$	46.43	0.28
	SR02	quercetin	$C_{15}H_9O_{7-}$	46.43	0.28
	SR03	stigmasterol	$C_{30}H_{50}$	43.83	0.76
	SR04	24-Ethylcholest-4-en-3-one	$C_{29}H_{48}O$	36.08	0.76
	SR05	poriferast-5-en-3beta-ol	$C_{29}H_{50}O$	36.91	0.75
	SR06	Sitosteryl acetate	$C_{31}H_{52}O_2$	40.39	0.85
Amomum villosum Lour. (Sharen)	SR07	beta-sitosterol	$C_{29}H_{50}O$	36.91	0.75
	SR08	icosa-11,14,17-trienoic acid methyl ester	$C_{21}H_{36}O_2$	44.81	0.23
	SR09	vitamin-e	$C_{24}H_{34}N_4O_5S$	32.29	0.7
	SR10	methyl icosa-11,14-dienoate	$C_{21}H_{38}O_2$	39.67	0.23
	SR11	(5S,8S,9S,10R,13R,14S,17R)-17-[(1R,4R)-4-ethyl-1,5-dimethylhexyl]-10,13-dimethyl-2,4,5,7,8,9,11,12,14,15,16,17-dodecahydro-1H-cyclopenta[a]phenanthrene-3,6-dione	$C_{29}H_{48}O_2$	33.12	0.79
	SR12	Stigmasta-5,22-dien-3-beta-yl acetate	$C_{31}H_{50}O_2$	46.44	0.86
Druce (Yuzhu)	YZ01	3-o-beta-d-glucopyranosyl-(1-2)-[beta-d-xylopyranosyl-(1-3)]-beta-d-glucopyranosyl-(1-4)-galactopyranosyl-25(S)-spirost-5(6)-en-3beta,14alpho-diol_qt	$C_{27}H_{42}O_4$	104.58	0.79
	YZ02	4',5,7-trihydroxy-6-methyl-8-methoxy-homoisoflavanone	$C_{18}H_{18}O_6$	89.7	0.33

YZ03	4',5,7-trihydroxy-6,8-dimethyl-homoisoflavanone	$C_{18}H_{18}O_5$	59.76	0.3
YZ04	polygosides E_qt	$C_{27}H_{36}O_3$	38.73	0.78
YZ05	4',5,7-trihydroxy-6-methyl-homoisoflavanone	$C_{17}H_{18}O_5$	82.94	0.27
YZ06	4'-methoxy-5,7-dihydroxy-6,8-dimethyl-homoisflavanone	$C_{19}H_{22}O_5$	57.14	0.34
YZ07	n-coumaroyltyramine	$C_{17}H_{17}NO_3$	85.63	0.2
YZ08	(Z)-3-(4-hydroxy-3-methoxy-phenyl)-N-[2-(4-hydroxyphenyl)ethyl]acrylamide	$C_{18}H_{19}NO_4$	118.35	0.26

Table S3 Target information of potential active ingredients of Maca compound preparations

No.	Uniprot ID	Target protein	Target gene	Frequency
1	P03372	Estrogen receptor alpha	ESR1	47
2	P30542	Adenosine A1 receptor	ADORA1	38
3	P29274	Adenosine A2a receptor (by homology)	ADORA2A	38
4	P12931	Tyrosine-protein kinase SRC	SRC	31
5	P23975	Norepinephrine transporter	SLC6A2	31
6	P37231	Peroxisome proliferator-activated receptor gamma	PPARG	31
7	P08185	Corticosteroid binding globulin	SERPINA6	29
8	P31645	Serotonin transporter	SLC6A4	29
9	P08581	Hepatocyte growth factor receptor	MET	28
10	P0DMS8	Adenosine A3 receptor	ADORA3	27
11	P33261	Cytochrome P450 2C19	CYP2C19	27
12	P04150	Glucocorticoid receptor	NR3C1	26
13	P42336	PI3-kinase p110-alpha subunit	PIK3CA	26
14	Q9UNQ0	ATP-binding cassette sub-family G member 2	ABCG2	24
15	P00533	Epidermal growth factor receptor erbB1	EGFR	24
16	Q16678	Cytochrome P450 1B1	CYP1B1	23
17	Q9Y233	Phosphodiesterase 10A	PDE10A	23
18	P11413	Glucose-6-phosphate 1-dehydrogenase	G6PD	23
19	P42345	Serine/threonine-protein kinase mTOR	MTOR	23
20	P27338	Monoamine oxidase B	MAOB	21
21	P10721	Stem cell growth factor receptor	KIT	21
22	P36888	Tyrosine-protein kinase receptor FLT3	FLT3	21
23	P28223	Serotonin 2a (5-HT2a) receptor (by homology)	HTR2A	20
24	P21397	Monoamine oxidase A	MAOA	19
25	Q9UM73	ALK tyrosine kinase receptor	ALK	19
26	O14965	Serine/threonine-protein kinase Aurora-A	AURKA	18
27	P00519	Tyrosine-protein kinase ABL	ABL1	18
28	P00734	Thrombin	F2	18
29	P14780	Matrix metalloproteinase 9	MMP9	17

30	P05177	Cytochrome P450 1A2	CYP1A2	17
31	P29275	Adenosine A2b receptor	ADORA2B	17
32	P42338	PI3-kinase p110-beta subunit	PIK3CB	16
33	O00329	PI3-kinase p110-delta subunit	PIK3CD	16
34	O14746	Telomerase reverse transcriptase	TERT	15
35	P14416	Dopamine D2 receptor (by homology)	DRD2	14
36	P11362	Fibroblast growth factor receptor 1	FGFR1	14
37	Q08499	Phosphodiesterase 4D	PDE4D	13
38	P07900	Heat shock protein HSP 90-alpha	HSP90AA1	12
39	P09619	Platelet-derived growth factor receptor beta	PDGFRB	12
40	P27986	PI3-kinase p85-alpha subunit	PIK3R1	12
41	P35462	Dopamine D3 receptor	DRD3	12
42	O60674	Tyrosine-protein kinase JAK2	JAK2	11
43	P11802	Cyclin-dependent kinase 4	CDK4	11
44	Q01959	Dopamine transporter	SLC6A3	11
45	O76074	Phosphodiesterase 5A	PDE5A	10
46	P60568	Interleukin-2	IL2	10
47	P31749	Serine/threonine-protein kinase AKT	AKT1	10
48	Q13946	Phosphodiesterase 7A	PDE7A	9
49	P36544	Neuronal acetylcholine receptor protein alpha-7 subunit	CHRNA7	9
50	P10415	Apoptosis regulator Bcl-2	BCL2	9
51	P08684	Cytochrome P450 3A4	CYP3A4	9
52	P15056	Serine/threonine-protein kinase B-raf	BRAF	9
53	Q06124	Protein-tyrosine phosphatase 2C	PTPN11	9
54	P42574	Caspase-3	CASP3	8
55	Q02750	Dual specificity mitogen-activated protein kinase kinase 1	MAP2K1	8
56	P11712	Cytochrome P450 2C9	CYP2C9	8
57	Q07343	Phosphodiesterase 4B	PDE4B	8
58	P21728	Dopamine D1 receptor	DRD1	8
59	P04049	Serine/threonine-protein kinase RAF	RAF1	8
60	P13500	C-C motif chemokine 2	CCL2	8
61	P15692	Vascular endothelial growth factor A	VEGFA	7
62	P78527	DNA-dependent protein kinase	PRKDC	7
63	P27815	Phosphodiesterase 4A	PDE4A	7
64	P08246	Leukocyte elastase	ELANE	7
65	P98170	Inhibitor of apoptosis protein 3	XIAP	7
66	O00408	Phosphodiesterase 2A	PDE2A	6
67	P01375	TNF-alpha	TNF	6
68	P05164	Myeloperoxidase	MPO	6
69	Q08493	Phosphodiesterase 4C	PDE4C	6
70	P40763	Signal transducer and activator of transcription 3	STAT3	6
71	O76083	Phosphodiesterase 9A	PDE9A	5

72	P41180	Calcium sensing receptor	CASR	5
73	P27169	Serum paraoxonase/arylesterase 1	PON1	5
74	Q06187	Tyrosine-protein kinase BTK	BTK	5
75	P07949	Kinesin-1 heavy chain/ Tyrosine-protein kinase receptor RET	RET	4
76	P41235	Hepatocyte nuclear factor 4-alpha	HNF4A	4
77	P22607	Fibroblast growth factor receptor 3	FGFR3	4
78	P08908	Serotonin 1a (5-HT1a) receptor	HTR1A	4
79	Q14432	Phosphodiesterase 3	PDE3A	4
80	Q13370	Phosphodiesterase 3B	PDE3B	4
81	P04626	Receptor protein-tyrosine kinase erbB-2	ERBB2	4
82	P36897	TGF-beta receptor type I	TGFBR1	4
83	P10635	Cytochrome P450 2D6	CYP2D6	4
84	Q14524	Sodium channel protein type V alpha subunit	SCN5A	3
85	P10276	Retinoic acid receptor alpha	RARA	3
86	O75874	Isocitrate dehydrogenase [NADP] cytoplasmic	IDH1	3
87	P00558	Phosphoglycerate kinase 1	PGK1	3
88	P04798	Cytochrome P450 1A1	CYP1A1	3
89	P35368	Alpha-1b adrenergic receptor	ADRA1B	3
90	O95263	Phosphodiesterase 8B	PDE8B	2
91	P16234	Platelet-derived growth factor receptor alpha	PDGFRA	2
92	P01112	Transforming protein p21/H-Ras-1	HRAS	2
93	P11217	Muscle glycogen phosphorylase	PYGM	2
94	P14222	Perforin-1	PRF1	2
95	P21964	Catechol O-methyltransferase	COMT	2
96	P46098	Serotonin 3a (5-HT3a) receptor (by homology)	HTR3A	2
97	P04062	Beta-glucocerebrosidase	GBA	2
98	P21802	Fibroblast growth factor receptor 2	FGFR2	2
99	P11166	Glucose transporter	SLC2A1	2
100	P19525	Interferon-induced, double-stranded RNA-activated protein kinase	EIF2AK2	2
101	Q9Y6K1	DNA (cytosine-5)-methyltransferase 3A	DNMT3A	1
102	Q13315	Serine-protein kinase ATM	ATM	1
103	Q9HCR9	Phosphodiesterase 11A	PDE11A	1
104	P19438	Tumor necrosis factor receptor R1	TNFRSF1A	1
105	P01137	Transforming growth factor beta-1	TGFB1	1
106	P29475	Nitric-oxide synthase, brain	NOS1	1
107	Q9HC29	Nucleotide-binding oligomerization domain-containing protein 2	NOD2	1
108	Q9Y2R2	Hematopoietic cell protein-tyrosine phosphatase 70Z-PEP	PTPN22	1
109	Q01064	Phosphodiesterase 1B	PDE1B	1
110	Q09472	Histone acetyltransferase p300	EP300	1
111	Q01432	AMP deaminase 3	AMPD3	1

112	P05362	Intercellular adhesion molecule-1	ICAM1	1
113	Q9Y5N1	Histamine H3 receptor	HRH3	1
114	P21554	Cannabinoid receptor 1	CNR1	1
115	Q99250	Sodium channel protein type II alpha subunit	SCN2A	1
116	P16499	Phosphodiesterase 6A	PDE6A	1
